# Reverse Shoulder Arthroplasty for Proximal Humeral Fractures: Is the Bigliani-Flatow Stem Suitable for Tuberosity Fixation and Healing?

**DOI:** 10.3390/jcm13123388

**Published:** 2024-06-09

**Authors:** Enrico Bellato, Valeria Fava, Andrea Arpaia, Michel Calò, Antonio Marmotti, Filippo Castoldi

**Affiliations:** 1 Department of Surgical Sciences, University of Turin, 10126 Turin, Italy; 2 San Luigi Gonzaga Hospital, 10043 Orbassano, Italy; 3 Orthopaedic and Trauma Unit, ASST-Sette Laghi, University of Insubria, 21100 Varese, Italy; vfava@studenti.uninsubria.it

**Keywords:** proximal humeral fractures, reverse total shoulder arthroplasty, Bigliani-Flatow stem, tuberosities’ healing

## Abstract

**Background/Objectives**: The aim of the study was to investigate the clinical, functional, and radiographic results of patients affected by three- or four-part proximal humeral fractures treated with reverse total shoulder arthroplasty, to investigate whether a prosthetic stem nonspecifically designed for fractures (i.e., the Bigliani-Flatow stem) promotes tuberosities’ healing, and to evaluate the impact of tuberosity fixation and healing on the outcomes. **Methods**: Patients’ data such as gender, age, side and dominancy, comorbidities, complications during or after surgery, and time lapse between trauma and surgery were prospectively collected. The type of fixation of the stem, the thickness and type of liner, and whether the tuberosities were fixed or not were also recorded. The Constant score weighted on the contralateral limb, QuickDASH, Oxford Shoulder Score, and Subjective Shoulder Value were collected. Tuberosities’ healing was assessed with X-rays (anteroposterior, Grashey, and axillary views). **Results**: Overall, 34 patients were included, with an average follow-up of 42 months. Tuberosities were reinserted in 24 cases and their healing rate was 83%. The mean values were the following: a Constant score of 64, Oxford Shoulder Score of 39, Subjective Shoulder Value of 71, and QuickDASH score of 27. There were no significant differences in the scores or range of motion between patients with tuberosities healed, reabsorbed, or not reattached. There was a better external rotation in the group with healed tuberosities and a longer duration of surgery to reattach tuberosities. **Conclusions**: The treatment of proximal humerus fractures with the Bigliani-Flatow stem is associated with good clinical and functional results. The healing rate of the tuberosities was high and comparable, if not even better, than the mean rates reported for the stems dedicated to fractures of the proximal humerus and was, therefore, also appropriate for this indication.

## 1. Introduction

Proximal humeral fractures are the third most common fractures of the upper extremity. They are mostly prevalent in the elderly and due to the population aging, the number is expected to increase worldwide [1,2,3,4,5,6,7,8].

Despite the high frequency and the number of studies, the choice of treatment is still one of the main controversies in the literature [9,10,11,12].

Conservative treatment represents the most frequent indication, since most of the fractures are not severely displaced, malunion can be well tolerated by the patient, and most of the time the patient is elderly with little functional expectations and is affected by comorbidities that might increase the risk of surgical complications [9,13,14,15]. In around 20% of the cases, surgical treatment is indicated with multiple fixation options available [14,16,17,18,19]. In a smaller percentage of cases, prosthetic replacement is needed due to fracture comminution. Hemiarthroplasty has lost popularity due to its unpredictable results [14,18,20,21,22], and is currently indicated in the rare scenario where the patient is relatively young with an unfixable fracture characterized by noncomminuted tuberosities.

Reverse total shoulder arthroplasty as a surgical choice for proximal humeral fractures is mainly indicated in elderly patients, in cases where the rotator cuff is deficient, or when tuberosities are comminuted. The surgical management of the tuberosities for reverse total shoulder arthroplasty in complex fractures is still debated; recent evidence seems to demonstrate that better outcomes can be observed if the tuberosities are reattached and healed [23,24,25,26,27,28,29]. Over the last years, companies have implemented their shoulder stem portfolio, introducing devices specifically aimed to treat proximal humeral fractures. Stems with a specific design for fractures are characterized by multiple holes to help tuberosity reattachment, fins to help the stability of the fixed tuberosities, rough surfaces to promote bone healing, and holes to better host the tuberosities or to provide room for a bone graft [30,31,32,33].

**Figure 1:**
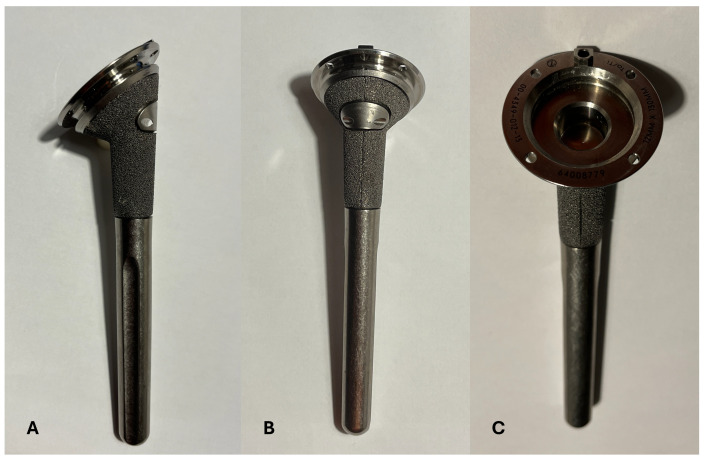
The Bigliani-Flatow stem. The stem is characterized by a proximal porous coating (**A**,**B**), a hole in the lateral side of the metaphyseal portion (**A**,**B**), and 4 holes in the most proximal portion (**C**).

The Bigliani-Flatow stem (Figure 1) is characterized by multiple diameters (from 8 to 16 mm) and lengths (130, 170, and 200 mm). It has a conical proximal part with an inlay configuration and a porous surface in tantalum trabecular metal that extends for 3.5 cm along the stem to facilitate bony integration and fixation. The metaphyseal portion is provided with a hole in the lateral side and four holes in the most proximal surface that can be used to fix the tuberosities. It can be used both in the cemented and uncemented configuration. It is convertible from reverse to hemiarthroplasty and hemiarthroplasty to reverse. The polyethylene liner can be either standard or retentive with three different thicknesses (+0, +3, +6 mm). In cases where at the end of the surgery, soft tissue tension and joint stability are not obtained with these three liners, an additional metal spacer can be added (+9 and +12 mm of thickness).

The Bigliani-Flatow stem has been reported in the literature regarding shoulder elective surgery [34,35,36,37,38], but few studies describing its use in patients affected by proximal humeral fractures are available [13,39,40,41].

The aim of the study was to investigate the clinical, functional, and radiographic results of patients affected by three- or four-part proximal humeral fractures treated with reverse total shoulder arthroplasty, to investigate whether a prosthetic stem nonspecifically designed for fractures (i.e., the Bigliani-Flatow stem) promotes tuberosities’ healing, and to evaluate the impact of tuberosity fixation and healing on the outcomes.

## 2. Materials and Methods

Patients affected by acute three- to four-part proximal humeral fractures or fracture-dislocations treated with reverse total shoulder arthroplasty between January 2015 and April 2021 were included in the study. Patients with a follow-up shorter than 1 year, patients treated with a prosthetic stem other than the 130 mm long Bigliani-Flatow stem (Zimmer Trabecular Metal Reverse Shoulder System—Zimmer, Warsaw, Indiana, USA), patients affected by pathological fracture, patients that resulted affected by postoperative infection, permanent nerve palsy, and periprosthetic fracture, and patients with comorbidities that would compromise the reliability of questionnaires (e.g., dementia, psychiatric conditions) were excluded.

The following data were prospectively collected: gender, age, side and dominancy, comorbidities, complications during or after surgery, time lapse between trauma and surgery, ASA score, type of anesthesia, glenosphere diameter, type of fixation of the stem, thickness and type of liner, and whether the tuberosities were fixed or not.

Postoperative shoulder flexion, abduction, and external rotation were measured using a long handheld goniometer. Internal rotation was approximated using a scoring system based on the Constant score. The vertebral level reached by the thumb was estimated visually and converted to the following scores: the lateral aspect of the thigh = 0, behind the buttock = 1, sacroiliac joint = 2, waist = 3, 12th thoracic vertebra = 4, and interscapular level = 5.

The clinical results were assessed with four different scores: the Constant score [42], the Quick Disability of the Arm, Shoulder, and Hand (DASH) [43], the Oxford Shoulder Score (OSS) [44], and the Subjective Shoulder Value (SSV) [45]. In the Constant score, 35 points are allocated to assess subjective impressions of daily life and pain and 65 points are allocated to assess objective measures such as range of motion and strength. The contralateral side was used to calculate the individual relative Constant score as described by Fialka et al. [46]. For patients with bilateral dysfunction, the individual relative Constant score was not calculated. In order to improve the reliability of the score, the modifications described by Blonna et al. were applied [47]. The strength was measured with a digital dynamometer (Brookstone, Merrimack, NH, USA). The QuickDASH is a shortened version of the DASH Outcome Measure and uses 11 items to subjectively measure physical function and symptoms in persons with any or multiple musculoskeletal disorders of the upper limb. The Oxford Shoulder Score is a 12-item patient-report questionnaire that contains two subscales, pain and activities of daily living. A 0–4 scoring format with a higher score representing better function was chosen [48]. The Subjective Shoulder Value is defined as a patient’s subjective shoulder assessment expressed as a percentage of an entirely normal shoulder, which would score 100%. Tuberosities’ healing was assessed with plain X-rays (anteroposterior, Grashey, and axillary views) that were analyzed using the software Telemis TM-PACS, version 4.94 (Louvain-la-Neuve, Belgium).

Surgeries were performed by a team of four shoulder surgeons. The patient was placed in the beach-chair position under general anesthesia. A systemic antibioprophylaxis with intravenous cefazolin (2 g) was administered. A double skin preparation [49] and a sterile drape were used. A deltopectoral approach was chosen in all the cases with the preservation of the cephalic vein when possible. The head fragment was removed using a tenaculum. For 3-part fractures, the lesser tuberosity was osteotomized from the head fragment. The tuberosities were first tagged using #2 sutures. Then, heavy braided non-resorbable sutures were passed through the rotator cuff at the osteotendinous junction to safely mobilize the tuberosities, and the glenoid was exposed. After glenoid component implantation (either Zimmer-Biomet Trabecular Metal or Zimmer-Biomet Comprehensive), the humerus was then approached with the arm in adduction and external rotation and progressively reamed. To restore humeral height, the medial calcar (if still intact) and upper margin of the pectoralis major tendon were used as landmarks. The stem was placed with 20° of retroversion. The thinnest inserts were used in all patients. If in between stem sizes or in cases where the metaphysis was comminuted, the stem was fixed using bone cement. In this case, care was taken to leave the proximal part of the stem covered with the trabecular metal implant without cement for tuberosities’ reinsertion. Tuberosities’ fixation was performed according to the surgeon’s intraoperative discretion using heavy braided non-resorbable sutures passed through two holes drilled at the lateral cortex of the diaphysis. A drain was inserted, and the surgical incision was closed. The patient wore a shoulder sling for pain relief for approximately 15 days after surgery and the mobilization was progressively allowed, regardless of the treatment of the tuberosities.

Descriptive statistics were reported as the average and standard deviation for continuous variables or frequency and percentage for categorical variables. The association between groups was performed using Chi-square for categorical variables and Mann–Whitney tests for qualitative variables. To study the difference in the scores of quantitative variables in variables with more than two modalities, the Kruskall–Wallis test was used. Statistical significance was defined as a *p*-value ≤ 0.05. Statistical analyses were performed using SPSS Statistics software (IBM SPSS Statistics for Windows, version 28.0; IBM Corp., Armonk, NY, USA).

The study was conducted according to the guidelines of the Declaration of Helsinki. In light of the Italian law, we are not required to ask for approval for this type of study. However, each author certifies that his or her institution has approved the human protocol for this investigation and that all investigations were conducted in conformity with ethical principles of research.

## 3. Results

**Figure 2:**
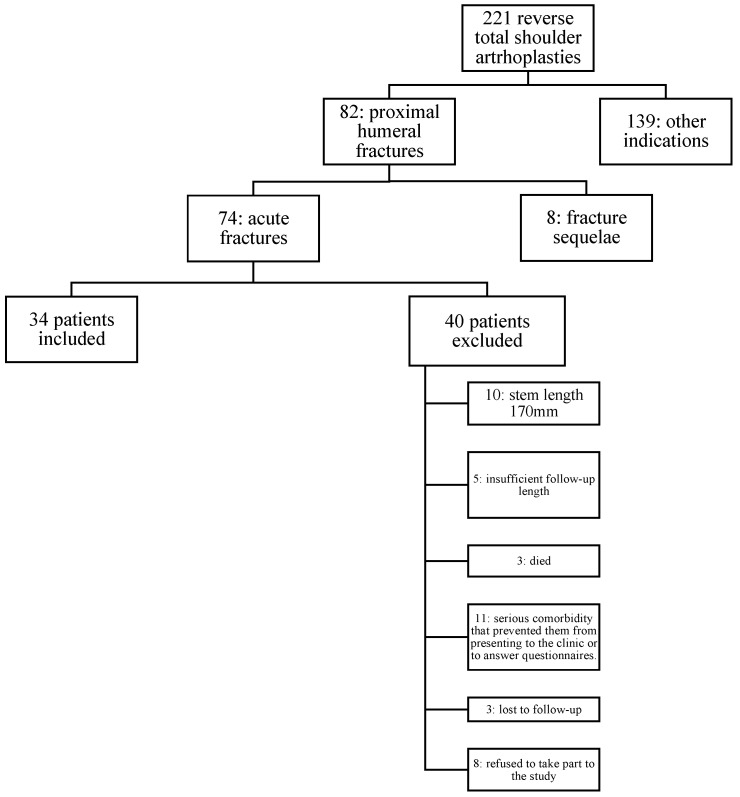
Flowchart portraying the inclusion process of the patients.

Among the 221 reverse total shoulder arthroplasties implanted in the index period, 34 patients fully met the inclusion criteria (Figure 2).

The average age at surgery was 71.2 ± 6.7 (57–84) years; 88.2% (30 patients) were women with an average age of 71.7 ± 6.6 (57–84), while 11.8% (4 patients) were men with an average age of 67 ± 6.4 (62–76). The mean follow-up length was 42.3 ± 20.9 (13–101) months.

The average surgical time was 112.8 ± 30.4 (72–230) minutes. Tuberosities were reattached in 24 out of 34 patients (70.6%); 83.3% (20 patients) of the reattached tuberosities healed (i.e., the resorption rate was 16.7%, 4 patients). The complication rate was 17.6%, but patients were affected only by minor complications; five patients required a blood transfusion, and one reported a transient radial nerve palsy.

A mean anterior elevation (EA) of 139.1° ± 28.1° (80°–180°), a mean abduction (ADB) of 128.5° ± 30.4 (80°–180°), and a mean external rotation with the adducted arm (ER1) of 4.2° ± 9.6 (0°–30°) were observed. As regards internal rotation (IR), 6.1% of patients managed to reach the interscapular level with their hand, 36.4% the 12th thoracic vertebra, 12.1% the waist, 27.3% the sacroiliac joint, and 18.2% could not go over the gluteus.

The mean Constant score was 64.4 ± 13.9 (34–87), with better results in the male population (*p* 0.006). The mean Modified Constant score was 0.84 ± 0.14 (0.42–1.06), the mean QuickDASH was 26.9 ± 22.2 (0–70.5), the mean OSS was 39.2 ± 7.7 (15–48), and the average SSV was 70.7 ± 23.1 (10–100) without any difference between male and female patients.

Patients were divided into two groups based on the treatment of the tuberosities (“not reattached” and “reattached”). These groups were homogeneous as no difference between them was found in terms of gender, age, comorbidities, type of anesthesia, affected side, diameter of the stem and its type of fixation, and glenosphere diameter and offset (*p* > 0.05). The functional scores and range of motion were not statistically different between these groups. However, a better trend in the scores and range of motion was observed in the “reattached” group, and the OSS score and ER1 were statistically significant better in this case (Table 1).

Also, the surgical duration was statistically different: 119 ± 33.3 (72–230) minutes versus 97.9 ± 14.7 (79–120) minutes (*p* 0.034) in the “reattached” and “not reattached” groups, respectively.

Patients were further divided into three groups based on tuberosity healing (“not reattached”, “reattached and healed”, “reattached and reabsorbed”). These groups were homogeneous. There was a statistically significant difference (*p* 0.028) regarding ER1 when tuberosities were reattached and healed (Table 2).

No other statistically significant difference was found in terms of the scores or range of motion between the groups, although a trend of better scores could be described in the group with the tuberosities healed. There was also a better trend in terms of EA, ADB, and IR in reattaching the tuberosities compared to not doing so, and a better EA and ABD in the group with the healed tuberosities compared to those with reabsorbed or non-reattached tuberosities.

Finally, no statistically significant difference was found in terms of the scores between the groups of patients with the groups “not reattached” and “reattached reabsorbed”.

## 4. Discussion

The use of reverse total shoulder arthroplasty for the treatment of complex three- and four-part humeral head fractures in the elderly has significantly increased in recent years, demonstrating satisfactory results, but with still several critical points and potential for improvement, especially regarding the internal and external rotation ranges [50,51,52,53,54]. The aim of this study was to investigate the clinical, radiological, and functional outcomes after reverse total shoulder arthroplasty in patients suffering from three- to four-part proximal humeral fractures or fracture-dislocations, and to analyze the role of tuberosity reinsertion on outcomes. Furthermore, it was investigated whether the Bigliani-Flatow stem, despite not having a specific design for fractures, promotes the healing of the tuberosities or not.

Studies that specifically evaluated the outcomes of reverse total shoulder arthroplasty for proximal humerus fractures reported a mean Constant score between 44 and 68, EA between 97° and 123°, ABD between 97° and 113°, and ER1 of approximately 18° to 25° [23,24,55,56,57]. These results are in line with the literature. Until some years ago, due to the lack of research, surgeons were somehow skeptical regarding the fixation of the tuberosities in reverse total shoulder arthroplasty; in fact, some authors did not fix the tuberosities [56] or even suggested their removal [23]. Even if recent studies have shown that tuberosity healing in older adults is associated with improved deltoid activation, especially during external rotation, and improved shoulder range of motion and function, tuberosity healing rates vary greatly, generally ranging from 40% to 84% in the elderly population [58]. In this study, the majority of the patients underwent the fixation of the tuberosities (70.6%), which healed in most of the cases (83.3%). Although the fixation of the tuberosities involves a significantly longer surgical time (119 min versus 97.9 min, *p* 0.034, in the present study) with the subsequent well-known drawbacks, the modern literature seems to support it. Boileau et al. [58] observed that the fixation and healing (84% of healing rate) of the tuberosity in reverse total shoulder arthroplasty for fractures improves anterior elevation, external rotation with an adducted limb, and patient satisfaction. Also, in the systematic review by Jain et al. [26], the authors reported improved range of motion and functional scores with healed tuberosities. In their systematic review, Anakwenze et al. [24] underlined that the fixation of the tuberosities is associated with better values of external rotation with the limb adducted and anterior elevation. In this study, only the ER1 was significantly better in patients with reinserted and healed tuberosities (*p* 0.02). No other statistically significant difference was found in terms of the scores or range of motion between the groups, although a trend of better scores could be described in the group with the tuberosities healed. There was also a better trend in terms of EA, ADB, and IR in reattaching the tuberosities compared to not doing so, and a better EA and ABD in the group with the healed tuberosities compared to those with reabsorbed or non-reattached tuberosities.

The Bigliani-Flatow stem used in this study has a conical proximal part with an inlay configuration and a porous surface in tantalum trabecular metal that extends for 3.5 cm. This allows for an initial stable fixation of the stem in the medullary canal, with subsequent eventual bony ingrowth. This seems to secure both the humeral shaft and tuberosities with long-term biologic fixation. Although it does not have a specific design for proximal humerus fractures, it has one hole on the lateral portion of the conical proximal part and four holes close to the circular surface where it hosts the liner. Also, compared to other more bulky stems with an inlay configuration (e.g., the Grammont-type stem), the volume of its proximal part is relatively small, and the back and lateral side of the stem can be filled with cancellous bone transplanted from the epiphysis [40]. This study demonstrated that the Bigliani-Flatow stem, despite not having a fracture-specific design, is associated with good clinical, functional, and radiographic outcomes and a high tuberosity healing rate, comparable to (and sometimes better than) the rates reported for stems dedicated to proximal humeral fractures [33,58,59]. As suggested by Wright et al. [39], the proximal conical flare of the stem might resist the superior displacement of the tuberosities when firmly secured with cerclage sutures and prevent the migration of the cerclage sutures and loss of fixation.

The porous surface of the prosthesis, which might explain the good results in terms of tuberosity healing, can also improve the fixation of the humeral component with no need of cementation [13,37,38,39,60,61]. Even the stem angle of inclination might play a role. Stems with a cervico-diaphyseal inclination of 145° (such as the Bigliani-Flatow stem) have been associated with a tuberosities’ healing rate of 69%, which is lower than 83% (in the 135° group) but higher than 66% (in the 155° group) [62].

In other clinical studies, the Bigliani-Flatow stem for proximal humeral fractures has been also associated with good outcomes. Wright et al. [39] prospectively reported on a cohort of 30 patients who underwent uncemented reverse total shoulder arthroplasty (using the Bigliani-Flatow stem along with either Zimmer Trabecular Metal or DJO Reverse Shoulder Prosthesis baseplates and glenospheres) as initial treatment for comminuted proximal humeral fractures. After a minimum follow-up of 12 months, the average American Shoulder and Elbow Surgeons Shoulder score was 82 ± 13.5, and the mean Simple Shoulder Test score was 69.4 ± 19.1%. The mean range of motion was 130° ± 31°of anterior elevation, 32° ± 18° of external rotation, and internal rotation to the mid-lumbar spine. In 70% of the cases, the tuberosities healed in an anatomical position, and in 97% of the cases, the X-rays showed a stable humeral stem fixation. In cases of metaphyseal comminution, the authors preferred to avoid cement and chose to use a longer stem; their results seem to support the use of an uncemented stem despite the risk of iatrogenic fracture. Chivot et al. [13], in their multicenter retrospective and comparative study, compared reverse total shoulder arthroplasty (Zimmer Trabecular Metal Reverse Shoulder System) with nonsurgical treatment in patients 70 years old or older with affected by proximal humeral fracture. Their results support the choice of shoulder replacement in independent elderly and healthy patients with a displaced fracture with three or four fragments. There was no significant difference in the mean scores of the QuickDASH, SSV, and visual analogue scale for pain, while the mean Constant score was significantly higher for the reverse total shoulder arthroplasty group (56.5 versus 50.5, *p* 0.03). The range of motion (in terms of anterior flexion, external rotation, and internal rotation) was significantly better in the reverse total shoulder arthroplasty group, which achieved a radiographic greater tuberosity union rate of 89%. The resorption rate was 10%. Tuberosities’ secondary displacement was only observed in the nonsurgical group with a rate of 12%. A recent retrospective study with a minimum follow-up of 2 years [40] compared 26 patients treated with the Bigliani-Flatow stem with 15 patients who underwent reverse total shoulder arthroplasty with a nonporous stem (Aequalis II, Wright Medical, Memphis, TN, USA). The functional scores (i.e., American Shoulder and Elbow Score and Constant score) were significantly higher in the first group. External rotation at the side was significantly better with the porous stem. The healing rate of the greater tuberosity in the first group was 88.5% compared to 46.7% in the second group (*p* 0.0082). The Bigliani-Flatow stem was used in the study published by Chalmers et al. [41], who prospectively compared reverse total shoulder arthroplasty (9 patients), hemiarthroplasty (9 patients), and open reduction and internal fixation (9 patients) in terms of both clinical and radiographic results and cost effectiveness. At a mean follow-up of 3 years, the three different treatment options were similar in terms of the subjective scores. Significantly more patients achieved >90° of active anterior elevation after reverse total shoulder arthroplasty, which also provided significant cost savings to Medicare.

The healing rate of the tuberosities in our study (83.3%) is in line with these studies that all show good outcomes.

Some studies have also reported better clinical and functional results when stems dedicated for proximal humerus fractures are used. Over the years, the design of the stem has been changed to promote tuberosity healing thanks to holes allowing stronger suture fixation or to metaphyseal “fins” reducing the rotational stress that hinders the healing process [30,31]. A recent meta-analysis [33] demonstrated that specific stems for fractures achieved a higher healing rate of the greater tuberosity (82.7%) compared to the standard stems (63.0%). These findings have been further supported by a recent systematic review and meta-analysis [32], which showed how dedicated stems are associated with significantly better scores and range of motion, and higher healing rates of the greater tuberosity. Despite the promising results of these studies, the healing rate in this study was high and comparable, and often even better, to the average rates reported for fracture-designed stems. However, it is worth saying that an important role is probably also played by the type and strength of the suture fixation of the tuberosities.

This study had some limitations. First, the number of patients included was limited. Second, only four male patients were included, but this female/male proportion is in line with the literature. Third, the length of follow-up was relatively short. Fourth, a control group is lacking. Fifth, the surgeries were performed by four different orthopedic surgeons, who performed tuberosities’ fixation without a consistent technique. However, the differences between the surgeons were little and the high healing rate of the tuberosities in these series seems to confirm that the fixation technique, even if not standardized, was valid. Sixth, a handheld goniometer was used for the range of motion assessment; a wearable motion capture system might provide more reliable data.

## 5. Conclusions

The treatment of proximal humerus fractures with the Bigliani-Flatow stem is associated with good clinical and functional results. The healing rate of the tuberosities was high and comparable, if not even better, to the mean rates reported for stems dedicated to fractures of the proximal humerus and was, therefore, also appropriate for this indication. Further studies are needed to define the superiority of reverse total shoulder arthroplasty-specific stems.

## Figures and Tables

**Table 1:** Descriptive statistics of the comparison between the two groups in terms of scores and range of motion.

	Not Reattached	Reattached	*p* Value
(n° of patients)	(10)	(24)	
Constant score	62.3	65.3	0.6
Modified Constant score	0.8	0.8	0.8
QuickDASH	32.7	24.5	0.4
Oxford Shoulder Score	35.6	40.7	0.06
SSV	0.7	0.7	0.5
EA	133°	141.7°	0.5
ABD	127.5°	128.9°	1
ER1	0°	7.3°	0.03
IR	2.3	3	0.2

**Table 2:** Descriptive statistics of the comparison between the three groups in terms of scores and range of motion.

	Not Reattached	Reattached, Healed	Reattached, Reabsorbed	*p* Value
(n° of patients)	(10)	(20)	(4)	
Constant score	62.3	65.4	64.5	0.8
Modified Constant score	0.8	0.8	0.9	1
QuickDASH	32.7	26.4	14.8	0.5
Oxford Shoulder Score	35.6	40.6	41.3	0.2
SSV	0.7	0.7	0.7	0.8
EA	133°	141.5°	142.5°	0.7
ABD	127.5°	131.3°	117.5°	0.6
ER1	0°	8.8°	0°	0.018
IR	2.3	3	3.3	0.3

## Data Availability

The data presented in this study are available on request from the corresponding author due to privacy reasons.

## References

[1] Borgström F., Karlsson L., Ortsäter G., Norton N., Halbout P., Cooper C. (2020). Fragility fractures in Europe: Burden, management and opportunities. Arch. Osteoporos..

[2] Sistema Nazionale per le Linee Guida dell’Istituto Superiore di Sanità (2021). Diagnosi, stratificazione del rischio e continuità assistenziale delle Fratture da Fragilità. https://siot.it/wp-content/uploads/2021/10/LG-392_Fratture-da-Fragilita.pdf.

[3] Curtis E.M., Moon R.J., Harvey N.C., Cooper C. (2017). Reprint of: The impact of fragility fracture and approaches to osteoporosis risk assessment worldwide. Int. J. Orthop. Trauma Nurs..

[4] Borgström F., Lekander I., Ivergård M., Ström O., Svedbom A., Alekna V., Bianchi M.L., Clark P., Curiel M.D., Dimai H.P. (2013). The International Costs and Utilities Related to Osteoporotic Fractures Study (ICUROS)—Quality of life during the first 4 months after fracture. Osteoporos. Int..

[5] Nuti R., Brandi M.L., Checchia G., Di Munno O., Dominguez L., Falaschi P., Fiore C.E., Iolascon G., Maggi S., Michieli R. (2019). Guidelines for the management of osteoporosis and fragility fractures. Intern. Emerg. Med..

[6] Pioli G., Bendini C., Pignedoli P., Giusti A., Marsh D. (2018). Orthogeriatric co-management—Managing frailty as well as fragility. Injury.

[7] IOF International Osteoporosis Foundation (2018). Ossa Spezzate, Vite Spezzate: Un Piano D’azione per Superare L’emergenza Delle Fratture da Fragilità in Italia.

[8] Chu S.P., Kelsey J.L., Keegan T.H.M., Sternfeld B., Prill M., Quesenberry C.P., Sidney S. (2004). Risk Factors for Proximal Humerus Fracture. Am. J. Epidemiology.

[9] Kancherla V.K., Singh A., Anakwenze O.A. (2017). Management of Acute Proximal Humeral Fractures. J. Am. Acad. Orthop. Surg..

[10] Murray I.R., Amin A.K., White T.O., Robinson C.M. (2011). Proximal humeral fractures: Current concepts in classification, treatment and outcomes. J. Bone Jt. Surg.-Ser. B.

[11] Neer C.S. (2006). THE CLASSIC: Displaced proximal humeral fractures: Part I. Classification and evaluation. 1970. Clin. Orthop. Relat. Res..

[12] Neer C.S. (2002). Four-segment classification of proximal humeral fractures: Purpose and reliable use. J. Shoulder Elb. Surg..

[13] Chivot M., Lami D., Bizzozero P., Galland A., Argenson J.-N. (2019). Three- and four-part displaced proximal humeral fractures in patients older than 70 years: Reverse shoulder arthroplasty or nonsurgical treatment?. J. Shoulder Elb. Surg..

[14] Handoll H.H., Elliott J., Thillemann T.M., Aluko P., Brorson S. (2022). Interventions for treating proximal humeral fractures in adults. Cochrane Database Syst. Rev..

[15] Rangan A., Handoll H., Brealey S., Jefferson L., Keding A., Martin B.C., Goodchild L., Chuang L.-H., Hewitt C., Torgerson D. (2015). Surgical vs. Nonsurgical Treatment of Adults With Displaced Fractures of the Proximal Humerus. JAMA.

[16] Gumina S., Candela V., Giannicola G., Orsina L., Passaretti D., Villani C. (2019). Complex humeral head fractures treated with blocked threaded wires: Maintenance of the reduction and clinical results with two different fixation constructs. J. Shoulder Elb. Surg..

[17] Euler S.A., Petri M., Venderley M.B., Dornan G.J., Schmoelz W., Turnbull T.L., Plecko M., Kralinger F.S., Millett P.J. (2017). Biomechanical evaluation of straight antegrade nailing in proximal humeral fractures: The rationale of the “proximal anchoring point”. Int. Orthop..

[18] Repetto I., Alessio-Mazzola M., Cerruti P., Sanguineti F., Formica M., Felli L. (2017). Surgical management of complex proximal humeral fractures: Pinning, locked plate and arthroplasty: Clinical results and functional outcome on retrospective series of patients. Musculoskelet. Surg..

[19] Blonna D., Assom M., Bellato E., Pisanu G., Greco V., Marmotti A., Rossi R. (2019). Outcomes of 188 Proximal Humeral Fractures Treated with a Dedicated External Fixator with Follow-up Ranging from 2 to 12 Years. J. Bone Jt. Surg..

[20] Shukla D.R., McAnany S., Kim J., Overley S., Parsons B.O. (2016). Hemiarthroplasty versus reverse shoulder arthroplasty for treatment of proximal humeral fractures: A meta-analysis. J. Shoulder Elb. Surg..

[21] Rajaee S.S., Yalamanchili D., Noori N., Debbi E., Mirocha J., Lin C.A., Moon C.N. (2017). Increasing Use of Reverse Total Shoulder Arthroplasty for Proximal Humerus Fractures in Elderly Patients. Orthopedics.

[22] van der Merwe M., Boyle M.J., Frampton C.M., Ball C.M. (2017). Reverse shoulder arthroplasty compared with hemiarthroplasty in the treatment of acute proximal humeral fractures. J. Shoulder Elb. Surg..

[23] Klein M., Juschka M., Hinkenjann B., Scherger B., Ostermann P.A.W. (2008). Treatment of Comminuted Fractures of the Proximal Humerus in Elderly Patients With the Delta III Reverse Shoulder Prosthesis. J. Orthop. Trauma.

[24] Anakwenze O.A., Zoller S., Ahmad C.S., Levine W.N. (2014). Reverse shoulder arthroplasty for acute proximal humerus fractures: A systematic review. J. Shoulder Elb. Surg..

[25] Saunders P.E., Walker J.B., Lederman E., McKee M.D.M. (2022). Current Role of Reverse Total Shoulder Arthroplasty for Fractures of the Proximal Humerus. J. Orthop. Trauma.

[26] Jain N.P., Mannan S.S., Dharmarajan R., Rangan A. (2018). Tuberosity healing after reverse shoulder arthroplasty for complex proximal humeral fractures in elderly patients—Does it improve outcomes? A systematic review and meta-analysis. J. Shoulder Elb. Surg..

[27] Russo R., Della Rotonda G., Cautiero F., Ciccarelli M. (2015). Reverse shoulder prosthesis to treat complex proximal humeral fractures in the elderly patients: Results after 10-year experience. Musculoskelet. Surg..

[28] Gallinet D., Adam A., Gasse N., Rochet S., Obert L. (2013). Improvement in shoulder rotation in complex shoulder fractures treated by reverse shoulder arthroplasty. J. Shoulder Elb. Surg..

[29] Ross M., Hope B., Stokes A., Peters S.E., McLeod I., Duke P.F. (2015). Reverse shoulder arthroplasty for the treatment of three-part and four-part proximal humeral fractures in the elderly. J. Shoulder Elb. Surg..

[30] Krishnan S.G., Reineck J.R., Bennion P.D., Feher L., Burkhead W.Z. (2011). Shoulder Arthroplasty for Fracture: Does a Fracture-specific Stem Make a Difference?. Clin. Orthop. Relat. Res..

[31] Li F., Zhu Y., Lu Y., Liu X., Wu G., Jiang C. (2014). Hemiarthroplasty for the treatment of complex proximal humeral fractures: Does a trabecular metal prosthesis make a difference? A prospective, comparative study with a minimum 3-year follow-up. J. Shoulder Elb. Surg..

[32] Onggo J.R., Nambiar M., Onggo J.D., Hau R., Pennington R., Wang K.K. (2021). Improved functional outcome and tuberosity healing in patients treated with fracture stems than nonfracture stems during shoulder arthroplasty for proximal humeral fracture: A meta-analysis and systematic review. J. Shoulder Elb. Surg..

[33] He S.-K., Liao J.-P., Guo J.-H., Huang F.-G. (2021). Fracture-Dedicated Prosthesis Promotes the Healing Rate of Greater Tuberosity in Reverse Shoulder Arthroplasty: A Meta-Analysis. Front. Surg..

[34] Mason R., Buckley T., Southgate R., Nicandri G., Miller R., Voloshin I. (2018). Radiographic Study of Humeral Stem in Shoulder Arthroplasty After Lesser Tuberosity Osteotomy or Subscapularis Tenotomy. Am. J. Orthop..

[35] Zmistowski B., Cahill S.V., Hill J.R., Gibian J.T., Sokrab R., Keener J.D., Aleem A.W. (2023). The rate and predictors of healing of repaired lesser tuberosity osteotomy in reverse total shoulder arthroplasty. JSES Int..

[36] Jobin C.M., Brown G.D., Bahu M.J., Gardner T.R., Bigliani L.U., Levine W.N., Ahmad C.S. (2012). Reverse total shoulder arthroplasty for cuff tear arthropathy: The clinical effect of deltoid lengthening and center of rotation medialization. J. Shoulder Elb. Surg..

[37] Wiater J.M., Moravek J.E., Budge M.D., Koueiter D.M., Marcantonio D., Wiater B.P. (2014). Clinical and radiographic results of cementless reverse total shoulder arthroplasty: A comparative study with 2 to 5 years of follow-up. J. Shoulder Elb. Surg..

[38] Bogle A., Budge M., Richman A., Miller R.J., Wiater J.M., Voloshin I. (2013). Radiographic results of fully uncemented trabecular metal reverse shoulder system at 1 and 2 years’ follow-up. J. Shoulder Elb. Surg..

[39] Wright J.O., Ho A., Kalma J., Koueiter D., Esterle J., Marcantonio D., Wiater J.M., Wiater B. (2019). Uncemented Reverse Total Shoulder Arthroplasty as Initial Treatment for Comminuted Proximal Humerus Fractures. J. Orthop. Trauma.

[40] Sasanuma H., Iijima Y., Saito T., Kanaya Y., Yano Y., Fukushima T., Nakama S., Takeshita K. (2020). Clinical results of reverse shoulder arthroplasty for comminuted proximal humerus fractures in elderly patients: A comparison between nonporous stems versus trabecular metal stems. JSES Int..

[41] Chalmers P.N., Slikker W., Mall N.A., Gupta A.K., Rahman Z., Enriquez D., Nicholson G.P. (2014). Reverse total shoulder arthroplasty for acute proximal humeral fracture: Comparison to open reduction–internal fixation and hemiarthroplasty. J. Shoulder Elb. Surg..

[42] Constant C.R., Murley A.G. (1987). A Clinical Method of Functional Assessment of the Shoulder. Clin. Orthop. Relat. Res..

[43] Beaton D.E., Wright J.G., Katz J.N. (2005). Development of the QuickDASH: Comparison of three item-reduction approaches. J. Bone. Joint. Surg. Am..

[44] Dawson J., Fitzpatrick R., Carr A.N.D.R.E.W. (1996). Questionnaire on the perceptions of patients about shoulder surgery. J. Bone. Joint. Surg. Br..

[45] Gilbart M.K., Gerber C. (2007). Comparison of the subjective shoulder value and the Constant score. J. Shoulder Elbow Surg..

[46] Fialka C., Oberleitner G., Stampfl P., Brannath W., Hexel M., Vécsei V. (2005). Modification of the Constant-Murley shoulder score-introduction of the individual relative Constant score Individual shoulder assessment. Injury.

[47] Blonna D., Scelsi M., Marini E., Bellato E., Tellini A., Rossi R., Bonasia D.E., Castoldi F. (2012). Can we improve the reliability of the Constant-Murley score?. J. Shoulder Elb. Surg..

[48] Dawson J., Rogers K., Fitzpatrick R., Carr A. (2009). The Oxford shoulder score revisited. Arch. Orthop. Trauma Surg..

[49] Blonna D., Allizond V., Bellato E., Banche G., Cuffini A.M., Castoldi F., Rossi R. (2018). Single *versus* Double Skin Preparation for Infection Prevention in Proximal Humeral Fracture Surgery. BioMed Res. Int..

[50] Federico S.G., Grassi A., Paladini P. (2018). Reverse Shoulder Arthroplasty Current Techniques and Complications.

[51] Grammont P.L.J.P., Trouilloud P., Laffay J.P., Deries X. (1987). Etude et réalisation d’une nouvelle prothèse d’épaule. Rhumatologie.

[52] Middernacht B., Van Tongel A., De Wilde L. (2016). A Critical Review on Prosthetic Features Available for Reversed Total Shoulder Arthroplasty. BioMed Res. Int..

[53] Huri G., Familiari F., Moon Y.L., Doral M.N., Muccioli G.M.M. (2019). Shoulder Arthroplasty: The Shoulder Club Guide.

[54] Roche C.P. (2022). Reverse Shoulder Arthroplasty Biomechanics. J. Funct. Morphol. Kinesiol..

[55] Bufquin T., Hersan A., Hubert L., Massin P. (2007). Reverse shoulder arthroplasty for the treatment of three- and four-part fractures of the proximal humerus in the elderly. J. Bone Jt. Surgery. Ser. B.

[56] Cazeneuve J.F., Cristofari D.-J. (2010). The reverse shoulder prosthesis in the treatment of fractures of the proximal humerus in the elderly. J. Bone Jt. Surgery. Ser. B.

[57] Marin R., Feltri P., Ferraro S., Ippolito G., Campopiano G., Previtali D., Surace M.F. (2023). Impact of tuberosity treatment in reverse shoulder arthroplasty after proximal humeral fractures: A multicentre study. J. Orthop Sci..

[58] Boileau P., Alta T.D., Decroocq L., Sirveaux F., Clavert P., Favard L., Chelli M. (2019). Reverse shoulder arthroplasty for acute fractures in the elderly: Is it worth reattaching the tuberosities?. J. Shoulder Elb. Surg..

[59] Garofalo R., Flanagin B., Castagna A., Lo E.Y., Krishnan S.G. (2015). Reverse shoulder arthroplasty for proximal humerus fracture using a dedicated stem: Radiological outcomes at a minimum 2 years of follow-up—Case series. J. Orthop. Surg. Res..

[60] Carpenter S.R., Urits I., Murthi A.M. (2016). Porous metals and alternate bearing surfaces in shoulder arthroplasty. Curr. Rev. Musculoskelet. Med..

[61] Kao D.S., Protzuk O.A., O’connell R.S. (2023). Clinical outcomes of cemented vs. uncemented reverse total shoulder arthroplasty for proximal humerus fractures: A systematic review. Eur. J. Orthop. Surg. Traumatol..

[62] O’Sullivan J., Lädermann A., Parsons B.O., Werner B., Steinbeck J., Tokish J.M., Denard P.J. (2020). A systematic review of tuberosity healing and outcomes following reverse shoulder arthroplasty for fracture according to humeral inclination of the prosthesis. J. Shoulder Elb. Surg..

